# Ergosterol Peroxide Inhibits Porcine Epidemic Diarrhea Virus Infection in Vero Cells by Suppressing ROS Generation and p53 Activation

**DOI:** 10.3390/v14020402

**Published:** 2022-02-15

**Authors:** Yi Liu, Xue Wang, Jing Wang, Jialu Zhang, Cong Duan, Jiufeng Wang

**Affiliations:** 1 College of Veterinary Medicine, China Agricultural University, Beijing 100193, China; ly2990571963@163.com (Y.L.); w792188860@126.com (X.W.); wangjing100193@163.com (J.W.); 18404969065@163.com (J.Z.); 2 China Institute of Veterinary Drug Control, Beijing 100193, China

**Keywords:** porcine epidemic diarrhea virus, ergosterol peroxide, life cycle, apoptosis, ROS, p53

## Abstract

Porcine epidemic diarrhea virus (PEDV) is an alphacoronavirus that causes severe watery diarrhea in piglets with high morbidity and mortality, resulting in serious economic losses to the farming industry. Ergosterol peroxide (EP) is a sterol with diverse biological activities including antiviral activity. In this study, we explored whether EP extracted from the fruiting body of the mushroom *Cryptoporus volvatus* had the potential to inhibit PEDV infection in Vero cells. The results revealed that EP had a remarkable inhibitory effect on PEDV infection. It could significantly inhibit multiple stages of the PEDV life cycle, including internalization, replication and release, and could directly inactivate PDCoV infectivity. However, it did not affect PEDV attachment. Furthermore, EP alleviated PEDV-induced apoptosis and mitigated the decrease in mitochondrial membrane potential caused by PEDV infection. It suppressed ROS generation and p53 activation caused by PEDV infection. The ROS scavenger N-acetyl-l-cysteine (NAC) and the p53 specific inhibitor Pifithrin-α (PFT-α) suppressed PEDV-induced apoptosis and impeded viral replication, suggesting that ROS and p53 play an important role in PEDV-induced apoptosis and viral replication. Collectively, EP can prevent PEDV internalization, replication and release, possesses the ability to directly inactivate PEDV, and can inhibit PEDV-induced apoptosis by interfering with PEDV-induced ROS production and p53 activation. These findings highlight the therapeutic potential of EP against PEDV infection.

## 1. Introduction

Porcine epidemic diarrhea virus (PEDV), a member of the family *Coronaviridae*, is one of the major viral pathogens causing porcine diarrhea, which can lead to a large number of morbidity and death in piglets [1]. PED has occurred in many countries since it was first reported in the UK in 1971 and is a severe problem endangering the pig industry at present [2,3]. Similar to other coronaviruses, PEDV is an enveloped virus that contains a single plus-stranded RNA genome of nearly 28 kb in length. It encodes two polymeric protein precursors (i.e., pp1a and pp1ab), four structural proteins (i.e., S, E, M, and N) and one accessory protein (ORF3) [4,5].

PEDV can induce apoptosis in a dose-dependent manner to benefit its replication [6]. It has been reported that PEDV mainly induces caspase-independent apoptosis by activating mitochondrial apoptosis-inducing factor [7]. Recent studies point out that PEDV infection activates caspase-8 and caspase-3, and apoptosis can be mediated through the activation of caspase-8 and caspase-3 in the late stage of PEDV infection [6,8]. Meanwhile, the p53–Puma (a proapoptotic factor) and reactive oxygen species (ROS)–p53 signaling pathways play an important role in mediating PEDV-induced apoptosis [9,10].

Tumor suppressor protein p53 plays a crucial role in the cell response to stress and participates in multiple biological processes, including cell cycle arrest and apoptosis [11]. It’s a transcriptional activator [12,13], and several of its targets are involved in mediating growth arrest and apoptosis [14]. Under normal physiological conditions, the expression level of p53 in cells is very low [15]. When cells undergo stresses, such as virus infection, p53 will be phosphorylated and transferred into the nucleus, which may lead to apoptosis [16]. In addition, the acetylation level of p53 is positively correlated with the activity of p53 [17,18]. ROS is another important factor that regulates apoptosis and is involved in several important apoptotic transduction pathways, such as p38 MAPK and p53 pathways [19]. Viral infection can trigger the overgeneration of ROS.

At present, there is no specific treatment for PEDV, and the current strategy is to reduce the risk of PEDV infection through vaccination [20]. The development of effective antiviral drugs is an important strategy to control PEDV infection. Ergosterol peroxide (EP), a sterol naturally present in medicinal mushrooms, lichens, sponges, etc., has been reported to exhibit bioactivities against neoplasms, inflammation and oxidation [21,22,23]. It is also an excellent natural antiviral compound with efficient inhibitory effects on several human and animal viruses [24]. We have previously found that EP can effectively inhibit porcine delta coronavirus (PDCoV) infection in vivo and in vitro [25,26,27]. Both PDCoV and PEDV belong to the family *Coronaviridae*. Therefore, it is necessary to study the effects of EP on PEDV infection and explore its potential antiviral mechanism.

Herein, we employed Vero cell models to evaluate the antiviral effects of EP against PEDV and elucidated the anti-PEDV mechanism of EP from the perspective of the virus life cycle and apoptosis.

## 2. Materials and Methods

### 2.1. Cell Culture and Viral Infection

Vero cells (ATCC CCL-81) were obtained from the American Type Culture Collection (ATCC) and cultured in Dulbecco’s modified Eagle’s medium/Nutrient Mixture F-12 (DMEM/F-12) (Gibco, Grand Island, NY, USA) supplemented with 10% heat-inactivated fetal bovine serum (FBS) (Gibco, Grand Island, NY, USA), 100 U/mL penicillin, and 100 μg/mL streptomycin. All cells were cultured in an incubator at 37 °C and 5% CO_2_.

The PEDV CV777 strain (GenBank accession no: AF353511) was used in the study. In all experiments, the cells were washed twice with serum-free medium before being infected with PEDV at a multiplicity of infection (MOI) of 0.5. After 1 h of PEDV adsorption, the cells were washed with serum-free medium for 2 times and then incubated at 37 °C before harvest.

### 2.2. Preparation of EP

EP was extracted from *Cryptoporus volvatus* with a purity of over 97%. Extraction and purity determination were performed as previously described [26].

### 2.3. Western Blot Analysis

The cells were lysed in radioimmunoprecipitation assay (RIPA) lysis buffer (ThermoFisher Scientific, Waltham, MA, USA) with 1 mM cocktail (ThermoFisher Scientific, USA) and 1 mM phenyl methyl sulfonyl fluoride (PMSF, Solarbio, Beijing, China) on ice. Protein concentrations were measured using the BCA Protein Assay Reagent (ThermoFisher Scientific, USA). Equivalent amounts of proteins were loaded and electrophoresed on 8–12% sodium dodecyl sulphate–polyacrylamide gel electrophoresis (SDS-PAGE). Subsequently, proteins were transferred to polyvinylidene difluoride (PVDF) membranes (Millipore Corp., Billerica, MA, USA). The membranes were blocked in phosphate-buffered saline (PBS) containing 0.1% (*v*/*v*) Tween 20 and 5% (*w*/*v*) bovine serum albumin (BSA) at room temperature for 1 h and then incubated overnight with indicated primary antibodies dissolved in BSA or non-fat milk at 4 °C. Primary antibodies were as follows: anti-β-actin (1:1000, Proteintech), anti-PEDV N (1:1000, Medgene Labs), anti-p-p53 (1:1000, Cell Signaling Technology), anti-Acetyl-p53 (1:1000, Cell Signaling Technology), anti-p53 (1:1000, Cell Signaling Technology), anti-Bax (1:1000, Abcam), anti-Bcl-2 (1:2000, Abcam), anti-Cleaved caspase-3 (1:2000, Abcam), and anti-caspase-3 (1:1000, Cell Signaling Technology). Thereafter, the membranes were incubated with corresponding horseradish peroxidase-conjugated secondary antibodies (1:5000, Proteintech) at room temperature for 1 h. The signal was detected using the ECL reagent (Millipore Corp., Billerica, MA, USA).

### 2.4. RNA Extraction and Quantitative Real-Time RT-PCR

Total RNA was extracted from Vero cells using RNAiso Plus (Takara, Tokyo, Japan) according to the manufacturer’s protocol. The specific primers for the PEDV-ORF3 gene, which encodes the non-structural accessory protein ORF3, were designed using the sequence of the PEDV CV777 (GenBank accession no. 935183) and Qrt-PCR was performed using the following specific primers: PEDV-ORF3-F: TATGCACTGTTTAAAGCCTCT, PEDV-ORF3-R: AGTAAATGCAGACTAAACAAAGCCT; GAPDH-F: GTGAAGGCTGAGAACGGGAA, GAPDH-R: AAATGAGCCCCAGCCTTCTC. The viral RNA levels were normalized by the GAPDH RNA level and the data were analyzed using the 2^−∆∆Ct^ method.

### 2.5. Indirect Immunofluorescence Assay (IFA)

Vero cells were washed twice with PBS and fixed with 4% paraformaldehyde for 15 min at 4 °C. The cells were permeabilized with 0.1% Triton X-100 on ice for 5 min. After washing 3 times with PBS, the cells were blocked with 3% BSA for 1 h at 37 °C. Subsequently, the cells were stained with an anti-PEDV monoclonal antibody (1:200, Medgene Lab, Brookings, SD, USA) at 4 °C for 12 h. The cells were then washed and incubated with Goat polyclonal Secondary Antibody to Rabbit IgG-H&L (Alexa Fluor^®^ 488) (1:200, Abcam, Cambridge, MA, USA) for 1 h at 37 °C. After 3 washes with PBS, the cells were incubated with DAPI (Sigma-Aldrich, St. Louis, MO, USA) at room temperature for 5 min and then examined with a fluorescence microscopy (Olympus CKX53, Tokyo, Japan).

### 2.6. Cytotoxicity Assay

The cytotoxicity of EP was assessed in Vero cells using the Cell Counting Kit-8 (CCK-8, DOJINDO, Tokyo, Japan) according to the manufacturer’s instructions. Vero cells were seeded into 96-cell plates and grown to 70% confluence for 24 h. After washing 3 times with PBS, the cells were treated with increasing concentrations of EP ranging from 50 to 200 Μm. Mock-treated cells were used as control. After 36 h, the cells were washed with PBS and incubated with 100 Μl DMEM/F12 and 10 Μl CCK-8 solution at 37 °C for 2 h. Absorbance was measured with a microplate reader (Infinite M200 PRO, TECAN, Hombrechtikon, Switzerland) at 450 nm.

### 2.7. Antiviral Assay

The cells were infected with PEDV (MOI = 0.5) in the absence or presence of EP (50, 100, or 200 Μm). After 1 h, the inoculum was removed, and the cells were washed 3 times with PBS. Subsequently, the cells were cultured in fresh medium in the absence or presence of EP (50, 100, or 200 Μm). The PEDV-infected cells were treated in the same manner with dimethyl sulfoxide (DMSO), the solvent for EP. At 24 h post-infection (hpi), viral replication was detected to assess the anti-PEDV activity of EP.

### 2.8. Viral Attachment, Internalization, Replication, Release, and Inactivation Assay

Viral attachment assay: The cells were incubated with PEDV (MOI = 0.5) in the absence or presence of EP (50 or 100 Μm) at 4 °C for 1 h. After washing 3 times with PBS, cell lysates were harvested for Qrt-PCR and fixed cells were used for IFA analysis.

Viral internalization assay: Vero cells were incubated with PEDV (MOI = 0.5) for 1 h at 4 °C. After the unbound viruses were removed by washing with PBS 3 times, the cells were incubated in DMEM/F-12 containing 2% FBS and different concentrations of EP at 37 °C for 1 h. Cell lysates were harvested for Qrt-PCR, and fixed cells were used for IFA analysis.

Viral replication assay: Vero cells were infected with PEDV (MOI = 0.5) for 1 h at 37 °C. To remove non-internalized viruses, the cells were washed with PBS (Ph 3.0). Cells were treated with DMEM/F-12 to remove the remaining PBS (Ph 3.0). Then, the cells were incubated with DMEM/F-12 containing 2% FBS and different concentrations of EP for 2 h at 37 °C. Cell lysates were harvested for Qrt-PCR, and fixed cells were used for IFA analysis.

Viral release assay: Vero cells were infected with PEDV (MOI = 0.5) for 10 h at 37 °C. The cells were then washed 3 times with PBS and incubated with DMEM/F-12 containing 2% FBS and different concentrations of EP at 37 °C for 12 h. The supernatants were harvested for Qrt-PCR, and the cells were fixed for IFA analysis.

Viral inactivation assay: The mixture of PEDV (MOI = 0.5) and EP (50 Μm or 100 Μm) was incubated at 37 °C for 2 h. The mixture was then washed with PBS and ultracentrifuged at 90,000× *g* for 1.5 h at 4 °C in 20% sucrose buffer (*w*/*w*) to purify the virus. Later, the virions were resuspended in the culture medium. Cell lysates were harvested for Qrt-PCR and fixed cells were used for IFA analysis [28].

### 2.9. Apoptotic Rate Measurement

Mock-infected or PEDV-infected cells were treated with different concentrations of EP. At 24 hpi, cell apoptosis was measured with the FITC Annexin V Apoptosis detection kit (Becton Dickinson Biosciences, San Diego, CA, USA) according to the manufacturer’s protocol. Briefly, cells were rinsed twice with PBS and re-suspended in 100 Μl binding buffer, followed by the addition of 5 Μl of Annexin V-FITC and 5 Μl of PI. After incubation in the dark for 15 min at 37 °C, 400 Μl of the binding buffer was added. Moreover, 10,000 cells were acquired, and the percentage of positive cells was analyzed by flow cytometry (BD FACSCalibur Flow Cytometer, BD Biosciences, San Diego, CA, USA).

### 2.10. Detection of Mitochondrial Membrane Potential (ΔΨm)

Mock-infected or PEDV-infected cells were treated with different concentrations of EP. At 24 hpi, the ΔΨm was detected using the mitochondrial membrane potential (ΔΨm) assay kit (Byotime, Shanghai, China) according to the manufacturer’s instructions. When the ΔΨm is high, JC-1 aggregates in the matrix of the mitochondria and forms a polymer (J-aggregates), which produces red fluorescence. When the ΔΨm is low, JC-1 cannot aggregate in the matrix of the mitochondria, and at this time, JC-1 is a monomer and produces green fluorescence. The ratio of aggregated JC-1 to monomeric JC-1 was used to estimate the ΔΨm. The fluorescence was visualized and photographed using a Leica SP8 Laser Scanning confocal microscope.

### 2.11. Determination of ROS Generation

Mock-infected or PEDV-infected cells were treated with different concentrations of EP. At 24 hpi, ROS accumulation was measured with the ROS assay kit (Byotime, China). Vero cells were harvested and suspended in PBS containing 10 mM DCFH-DA at 37 °C for 20 min in the dark. After the cells were washed with serum-free medium 3 times, ROS levels were detected by flow cytometry.

### 2.12. Inhibitor Treatments

The ROS scavenger N-acetyl-l-cysteine (NAC) (10 μM) and the p53 specific inhibitor Pifithrin-α (PFT-α) (10 μM) were added to Vero cells 1 h before infection. Whereafter, the inoculum was removed, and the cells were infected with PEDV (MOI = 0.5). After 1 h of PEDV adsorption, the virus inoculum was removed and fresh medium containing inhibitors was added to the cells. At 24 hpi, the cells were used for the detection of related indices.

### 2.13. Statistical Analysis

All experiments were performed 3 times. Differences between the means were compared using Tukey’s honestly significant difference post hoc test. The data were visualized using GraphPad Prism 8 software (GraphPad Software, San Diego, CA USA) and expressed as the mean ± SEM. The statistically significant differences were set at *p* < 0.05.

## 3. Results

### 3.1. Ergosterol Peroxide Inhibited PEDV Infection in Vero Cells

**Figure 1:**
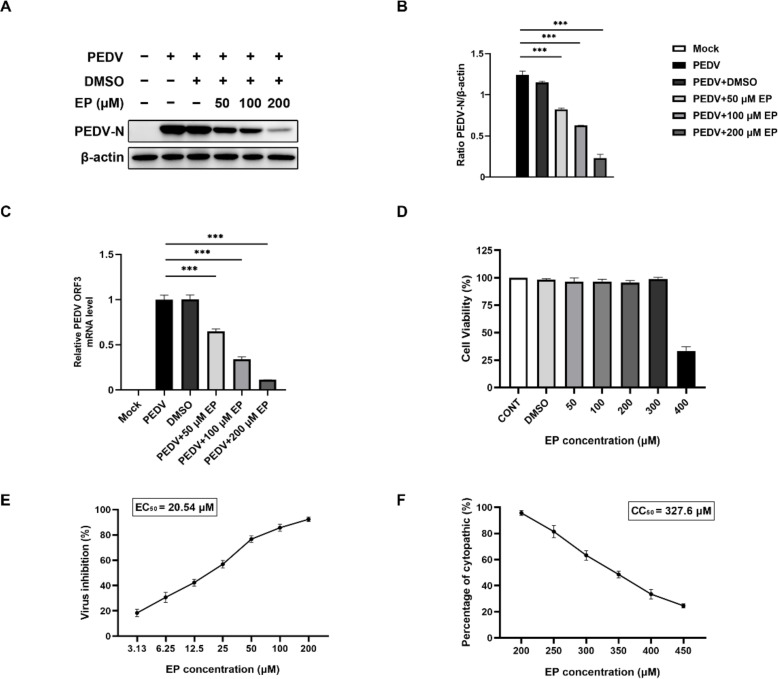
Ergosterol peroxide (EP) inhibits PEDV infection in Vero cells. Vero cells were mock-infected or infected with PEDV (MOI = 0.5) in the absence or presence of EP. After 1 h, the cells were further cultured in fresh medium in the absence or presence of EP. At 24 h post infection (hpi), the cells were detected for the related indices. (**A**) The expression of PEDV N was analyzed by Western blot. (**B**) Results were presented as the ratio of PEDV N band intensity to β-actin band intensity. (**C**) The relative viral RNA level was analyzed by qRT-PCR. (**D**) Determination of cytotoxicity of EP by CCK-8 assay. (**E**) EC_50_ of EP against PEDV infection was checked. (**F**) CC_50_ of EP on Vero cells was checked. Values represent the mean ± SEM for three independent experiments. *** *p* < 0.001.

To assess the antiviral activity of EP against PEDV, the expression level of PEDV N protein in Vero cells was first evaluated by Western blot. The results showed that EP reduced the expression of PEDV N protein in a dose-dependent manner, with a reduction rate of 30.1–82.6% (Figure 1A,B). The PEDV ORF3 mRNA level was then assessed by qRT-PCR to confirm the inhibitory effects of EP on PEDV replication. As expected, the PEDV ORF3 mRNA level was reduced significantly in PEDV-infected cells treated with different concentrations of EP (Figure 1C). The cytotoxicity of EP on Vero cells was evaluated by CCK-8 assay. The results showed that treatment of Vero cells with 50, 100, 200, or 300 μM EP for 24 h had no significant effect on cell viability (Figure 1D). Meanwhile, we calculated the half maximal effective concentration (EC_50_) and the half maximal cytopathic concentration (CC_50_) of EP (Figure 1E,F). The selective index of the drug (SI) was 15.95.

### 3.2. The Effect of EP on Different Stages of the PEDV Life Cycle in Vero Cells

**Figure 2:**
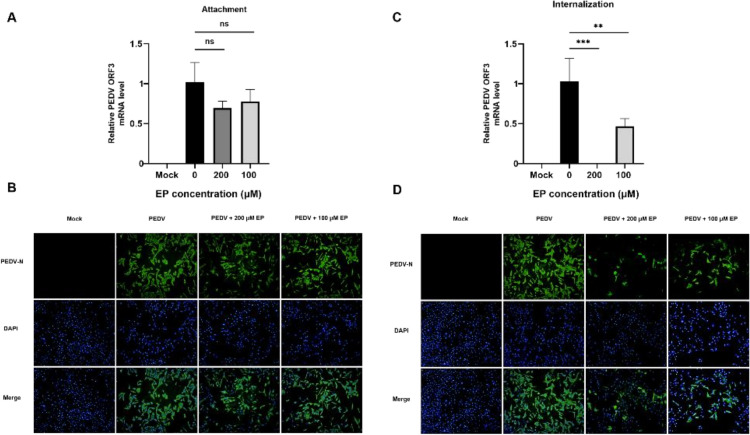
The effect of EP on PEDV attachment and internalization in Vero cells. Mock-infected or infected cells were treated with different concentrations of EP during viral attachment stage. PEDV replication was determined by qRT-PCR (**A**) and IFA (**B**). Mock-infected or infected cells were treated with different concentrations of EP during viral internalization stage. PEDV replication was determined by qRT-PCR (**C**) and IFA (**D**). Green fluorescence represents the PEDV distribution, and blue fluorescence represents the nuclear distribution. Values represent the mean ± SEM for three independent experiments. ** *p* < 0.01; *** *p* < 0.001.

**Figure 3:**
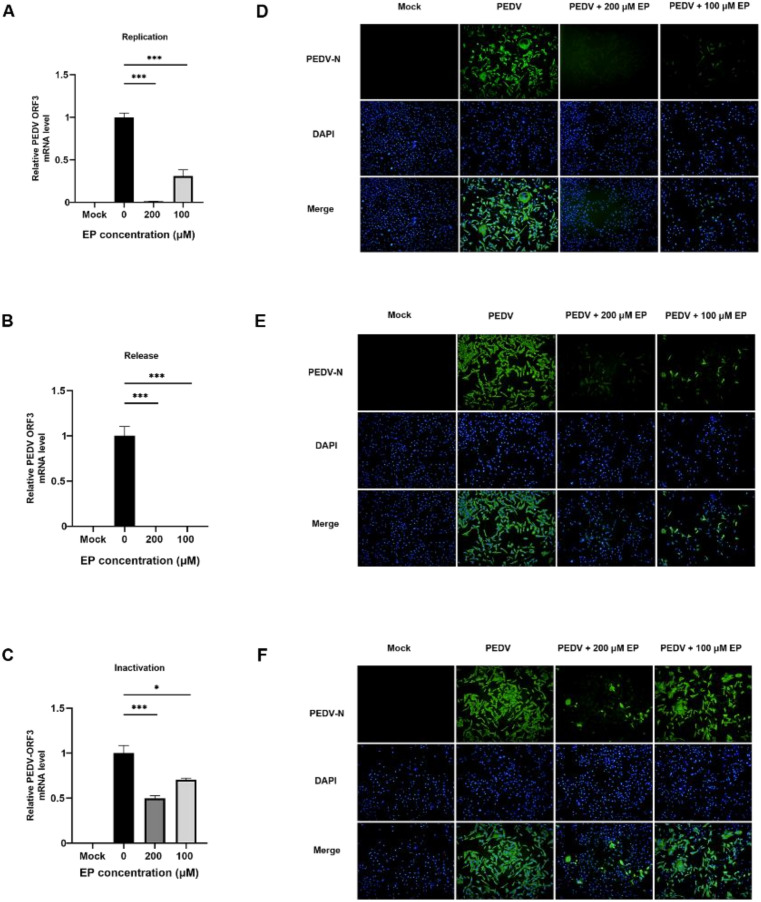
The effect of EP on PEDV replication, release and virions in Vero cells. Mock-infected or infected cells were treated with different concentrations of EP during viral replication stage. PEDV replication was determined by qRT-PCR (**A**) and IFA (**D**). Mock-infected or infected cells were treated with different concentrations of EP during viral release stage. PEDV replication was determined by qRT-PCR (**B**) and IFA (**E**). The direct inactivation of EP on PEDV virions was determined by qRT-PCR (**C**) and IFA (**F**). Green fluorescence represents the PEDV distribution, and blue fluorescence represents the nuclear distribution. Values represent the mean ± SEM for three independent experiments. * *p* < 0.05; *** *p* < 0.001.

In the viral attachment assay, there was no significant difference in the level of PEDV ORF3 mRNA and the number of PEDV-positive cells between EP-treated and non-EP-treated infected cells, implying that EP did not affect viral attachment to Vero cells (Figure 2A,B). However, they dramatically decreased in an EP dose-dependent manner when the infected cells were treated with EP during the stage of viral internalization (Figure 2C,D), replication (Figure 3A,D) or release (Figure 3B,E), suggesting that EP has inhibitory effects on PEDV internalization, replication and release. Besides, viral inactivation assays were performed to determine whether EP had any direct effect on PEDV virions. The results showed that the infectivity of PEDV was obviously attenuated, indicating that EP can directly inactivate PEDV virions (Figure 3C,F).

### 3.3. Ergosterol Peroxide Alleviated PEDV-Induced Apoptosis in Vero Cells

**Figure 4:**
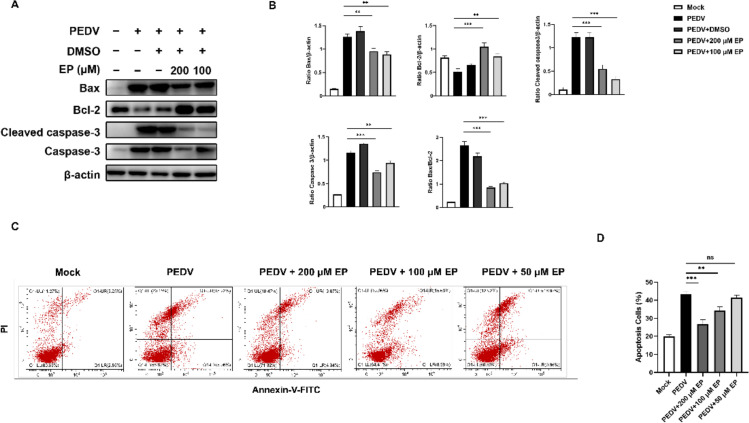
EP inhibits PEDV-induced apoptosis in Vero cells. Mock-infected or PEDV-infected cells were treated with different concentrations of EP. At 24 hpi, the cells were detected for the related indices. (**A**) The expressions of Bax, Bcl-2, cleaved caspase-3, and caspase-3 were analyzed by western blot. (**B**) Results were presented as the ratio of target protein band intensity to β-actin band intensity. (**C**,**D**) The apoptosis rates were analyzed by flow cytometry. Values represent the mean ± SEM for three independent experiments. ** *p* < 0.01; *** *p* < 0.001.

PEDV-induced apoptosis is beneficial to viral replication and cytopathic effects (CPE). Here, we investigated whether EP could inhibit PEDV-induced apoptosis by western blot and flow cytometry. PEDV infection increased pro-apoptotic Bax expression, facilitated the cleavage of the apoptotic executioner caspase-3 and decreased anti-apoptotic Bcl-2 expression (Figure 4A,B). EP treatment reversed these phenomena (Figure 4A,B). FITC-labeled Annexin V is commonly used to specifically target and identify apoptotic cells. The positive rate of Annexin V in infected cells treated with EP decreased in a dose-dependent manner compared with that in infected cells without EP (Figure 4C,D). These data confirm that EP has an inhibitory effect on PEDV-induced apoptosis.

### 3.4. Ergosterol Peroxide Alleviates the Decrease in ΔΨm Caused by PEDV Infection

**Figure 5:**
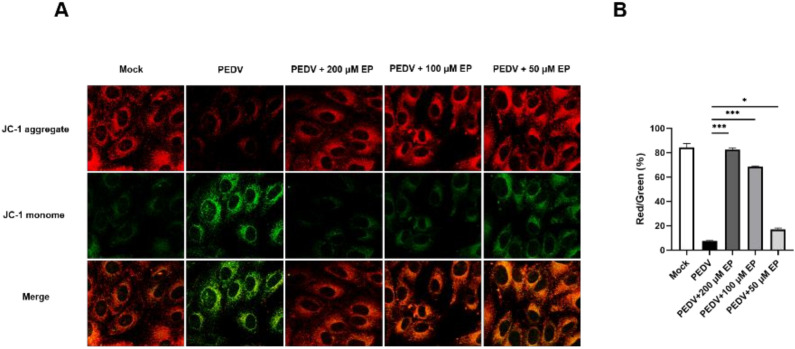
EP alleviates the decrease in mitochondrial membrane potential (ΔΨm) caused by PEDV infection. (**A**) Mock-infected or PEDV-infected cells were treated with different concentrations of EP. At 24 hpi, the cells were detected for the ΔΨm by JC-1 fluorescence staining. (**B**) Results were presented as the ratio of JC-1 aggregate (red fluorescence) to JC-1 monome (green fluorescence). JC-1 aggregate (red fluorescence) indicates high ΔΨm whereas JC-1 monome (green fluorescence) indicates low ΔΨm Values represent the mean ± SEM for three independent experiments. * *p* < 0.05; *** *p* < 0.001.

Mitochondrial apoptotic pathway plays a central role in PEDV-induced apoptosis. The decrease in ΔΨm is a landmark event in the early stage of apoptosis. The ratio of aggregated JC-1 (red fluorescence) to monomeric JC-1 (green fluorescence) is commonly used to represent the ΔΨm and reflect the degree of mitochondrial depolarization. As shown in Figure 5, a marked decrease in the ratio of red to green fluorescence was observed in PEDV-infected cells, suggesting that PEDV infection results in the decrease in ΔΨm and causes mitochondrial dysfunction. However, when the infected cells were treated with EP, the ΔΨm was restored evidently. These results indicate that EP can inhibit PEDV-induced apoptosis in Vero cells through alleviating PEDV-induced mitochondrial dysfunction.

### 3.5. Ergosterol Peroxide Inhibited p53 Activation and ROS Generation Induced by PEDV Infection

**Figure 6:**
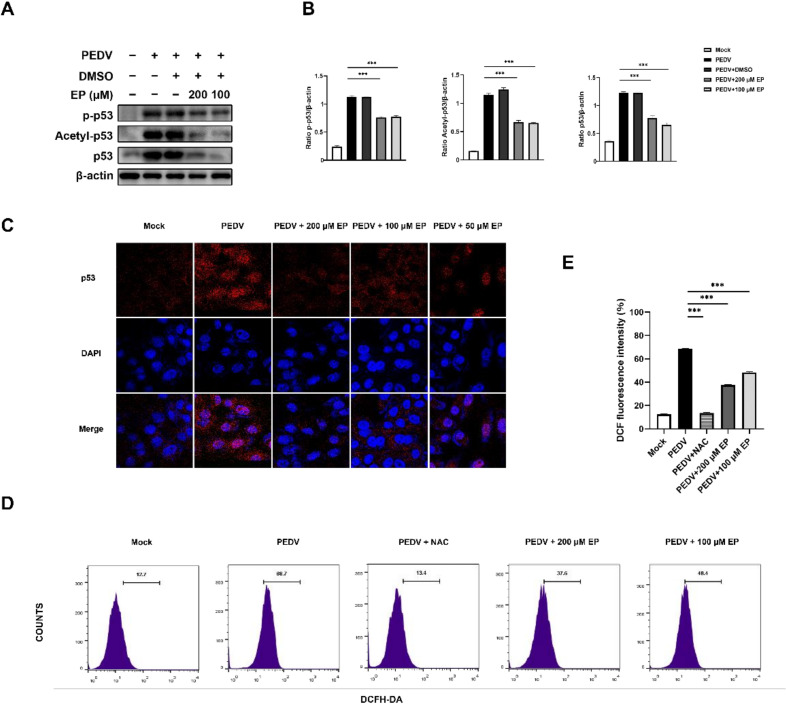
EP inhibits p53 activation and ROS generation induced by PEDV infection in Vero cells. Mock-infected or PEDV-infected cells were treated with different concentrations of EP or 10 μM N-acetyl-l-cysteine (NAC). At 24 hpi, the cells were detected for the related indices. (**A**) The expressions of p-p53, Acetyl-p53, and p53 were analyzed by Western blot. (**B**) Results were presented as the ratio of target protein band intensity to β-actin band intensity. (**C**) Immunofluorescence analysis of intracellular p53 protein levels. (**D**,**E**) The ROS level was evaluated by analyzing DCF fluorescence intensity by flow cytometry. Values represent the mean ± SEM for three independent experiments. *** *p* < 0.001.

Considering that p53 and ROS are important mediators of PEDV-induced apoptosis, we examined the influence of EP on p53 activity and ROS generation during PEDV infection. Western blot analysis revealed an increase in the expressions of p53, p-p53, and acetyl-p53 in the PEDV-infected cells, indicating that p53 was activated by PEDV infection (Figure 6A,B). Treatment with EP mitigated this increase (Figure 6A,B). The subcellular localisation of p53 further verified the above results. In PEDV-infected cells, a large amount of p53 gathered in the nucleus (Figure 6C). EP treatment attenuated p53 nuclear translocation caused by PEDV infection (Figure 6C).

PEDV infection induced an increase in DCF fluorescence in Vero cells, indicating that PEDV infection elevated ROS levels (Figure 6D,E), while EP treatment significantly reduced DCF fluorescence (Figure 6D,E).

### 3.6. Ergosterol Peroxide Reduces PEDV-Induced Apoptosis by Inhibiting ROS Generation and p53 Activation

**Figure 7:**
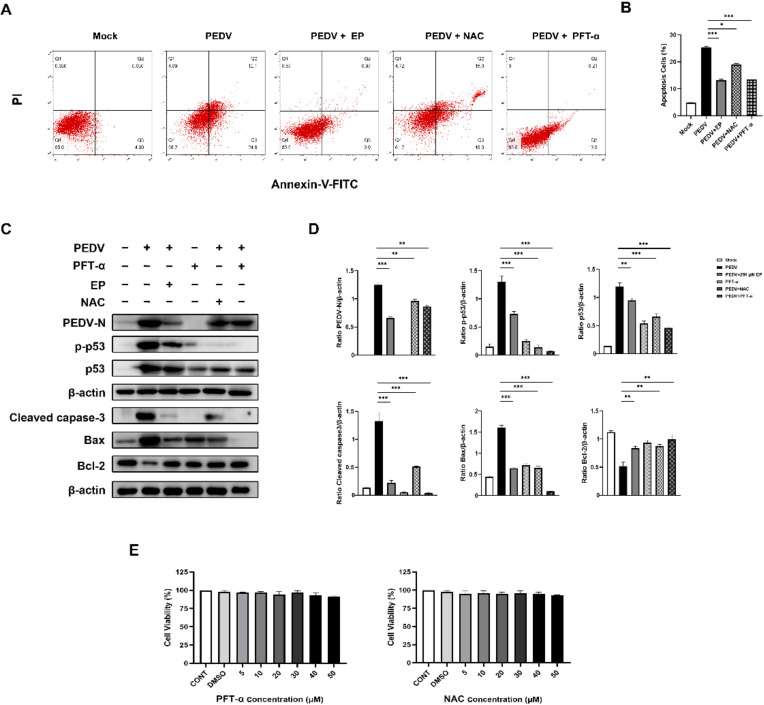
EP reduces PEDV-induced apoptosis by inhibiting ROS generation and p53 activation. Mock-infected or PEDV-infected cells were treated with different concentrations of EP, 10 μM NAC or 10 μM Pifithrin-α (PFT-α). At 24 hpi, the cells were detected for the related indices. (**A**,**B**) The apoptosis rates were analyzed by flow cytometry. (**C**) The expressions of PEDV N, p-p53, p53, cleaved caspase3, Bax, and Bcl-2 were analyzed by Western blot. (**D**) Results were presented as the ratio of target protein band intensity to β-actin band intensity. (**E**) Determination of cytotoxicity of NAC and PFT-α by CCK-8 assay. Values represent the mean ± SEM for three independent experiments. * *p* < 0.05; ** *p* < 0.01; *** *p* < 0.001.

To further clarify the mechanism of EP alleviating PEDV-induced apoptosis from the perspective of p53 and ROS, we treated Vero cells with the p53 specific inhibitor PFT-α and the ROS scavenger NAC, respectively, and then analyzed the rate of apoptosis and the expressions of apoptosis-related proteins. Flow cytometry analysis demonstrated that both EP and PFT-α significantly reduced the percentage of apoptotic cells in infected cells (Figure 7A,B). Western bolt analysis showed that NAC and PFT-α decreased the expressions of Bax and cleaved caspase-3, increased Bcl-2 expression, and reduced PEDV N expression in infected cells (Figure 7C,D), indicating that ROS and p53 play a pivotal role in PEDV-induced apoptosis and viral replication. Besides, NAC inhibited p53 phosphorylation during PEDV infection (Figure 7C,D), uncovering that ROS as the upstream signal of p53 mediates PEDV-induced apoptosis. EP inhibited ROS generation (Figure 6D,E) and reduced the expressions of p53 and PEDV N (Figure 7C,D), suggesting that EP can inhibit PEDV-induced apoptosis by inhibiting ROS generation and p53 activation, thereby inhibiting PEDV replication. The treatment concentration of PFT-α and NAC had no toxic effects on Vero cells (Figure 7E).

## 4. Discussion

Our previous studies confirmed that EP can effectively inhibit PDCoV infection in vitro and in vivo [20,26]. Here, we carried out an in vitro study to evaluate the inhibitory effect of EP on PEDV infection and found that EP possesses an outstanding anti-PEDV activity.

We then conducted experiments to characterize the anti-PEDV mechanism of EP. The antiviral mechanisms of antiviral agents can be divided into two major types: (i) against the virus itself; (ii) against the host factors involved in viral infection. Viral attachment, internalization, replication and release are important parts of the coronavirus life cycle. Perturbing any of these processes will suppress viral infection. Our results demonstrated that EP could interfere with multiple steps of the PEDV life cycle, including internalization, replication and release. Internalization is a crucial early step in the coronavirus life cycle. Intervention strategies targeting viral internalization are particularly effective in preventing viral infection and accompanying inflammatory diseases. Our previous study found that EP can also inhibit the internalization of PDCoV [20,26]. The S protein of coronavirus mediates viral entry into cells by first binding to cellular receptors through the S1 subunit and then fusing host and viral membranes through the S2 subunit [29]. Studies have shown that the PDCoV S2 subunit is structurally similar to α- and β-coronavirus S2 subunits, and it is inferred that coronaviruses rely on a common fusion mechanism to enter host cells [29,30]. Therefore, we speculated that EP might interpose virus-cell membrane fusion of PEDV and PDCOV through the same mechanism, thus inhibiting viral internalization. This speculation should be validated with more α- and β-coronaviruses. Coronaviruses encode multiple proteases and replicases, such as 3C-like protease (3CLpro), papain-like protease (PLpro) and RNA-dependent RNA polymerase (RdRp), that play a crucial role in viral replication. EP may perturb the synthesis of PEDV RNA and protein by affecting the activity of these enzymes. Moreover, EP could inactivate PEDV directly. It may disrupt the integrity of virions, which is necessary to prevent the viral genome from being exposed to RNase and being degraded, so as to exert its viricidal effects.

PEDV mainly infects the small intestine. The intestinal barrier serves two important functions: first, it allows nutrients absorption; Second, it protects the body from harmful substances and pathogens from the external environment. Intestinal epithelial homeostasis which is established by equilibrium between cell proliferation and cell death is one of the keys to maintaining the integrity of intestinal barrier. PEDV has been confirmed to induce apoptosis in vitro and in vivo [20,31]. This may lead to the destruction of the intestinal barrier, thus affecting the absorption of nutrients and water, and causing diarrhea. In addition, apoptosis contributes to virus-induced CPE in vitro and/or tissue damage in vivo [27]. Herein, we observed that PEDV-induced apoptosis was alleviated by EP treatment. Considering that some viruses trigger apoptosis to facilitate the dissemination of viral progeny, we speculate that the inhibitory effect on PEDV-induced apoptosis might be part of the mechanism by which EP inhibits PEDV release. Thereafter, we explored the mechanism of EP alleviating PEDV-induced apoptosis from the perspective of p53 and ROS.

p53 transactivates key molecules in several apoptotic pathways, including Fas, Puma, Noxa, and Bax, and the p53 apoptotic pathway is a classical apoptotic mechanism [32]. PEDV infection can activate the proapoptotic factor Puma and induce apoptosis in a p53-dependent pathway [10]. In this study, we found that PEDV infection activates p53, and the p53 inhibitor PFT-α inhibits PEDV-induced apoptosis and hampered PEDV replication, implicating that PEDV infection can trigger apoptosis by activating the p53 pathway, thereby promoting virus replication [7]. EP can inhibit PEDV-induced p53 activation, which constitutes its mechanism to relieve PEDV-induced apoptosis and inhibit virus replication.

p53 can be regulated by ROS during PEDV infection [9]. ROS is a by-product of metabolism and plays an important role in cell signal transduction and homeostasis [33]. It participates in many cellular processes such as cell proliferation, apoptosis and inflammation. ROS accumulation directly caused by viruses or host immune responses is a potentially important pathogenic mechanism. Many viral infections promote ROS generation, which is an important factor in the occurrence of virus-induced apoptosis, such as transmissible gastroenteritis virus, another enteropathogenic α-coronavirus [34]. In this study, we found that the ROS scavenger NAC inhibited p53 activation and decreased the expression of apoptotic proteins, ascertaining that ROS acts as an upstream signal of p53 to mediate the PEDV induced apoptosis. EP can mitigate ROS generation caused by PEDV.

Intriguingly, the inhibitory effect of EP on PEDV replication was evidently stronger than that of NAC and PFT-α, implicating that EP also inhibits PEDV infection through other pathways that we have not studied here. We previously found that EP can inhibit PDCoV replication by alleviating PDCoV-induced autophagy [25]. PEDV infection can induce autophagy through ROS mediated endoplasmic reticulum stress, and this process enhances viral replication [35]. Hence, EP may also inhibit PEDV replication by suppressing PEDV-induced autophagy.

**Figure 8:**
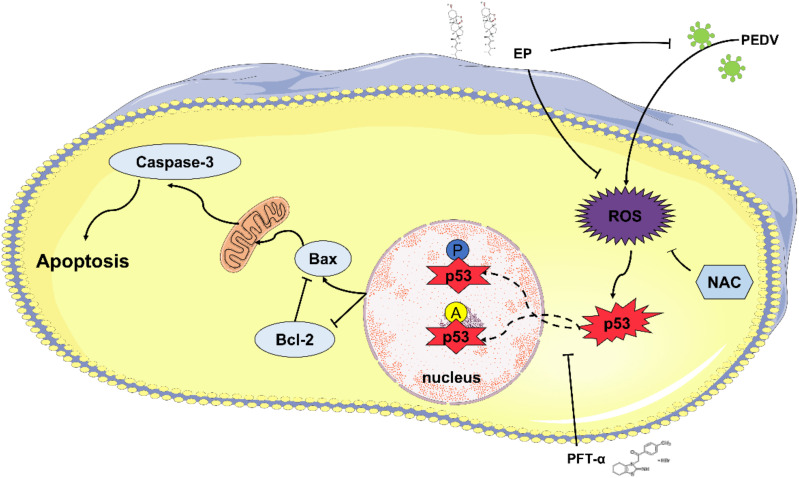
Schematic diagram of EP inhibiting PEDV-induced ROS/p53-dependent apoptosis. PEDV infection increases ROS generation, promotes p53 phosphorylation and nuclear translocation, and then upregulates Bax expression and downregulates Bcl-2 expression. Finally, it leads to caspase-3 activation and induces apoptosis. EP can reduce ROS accumulation and alleviate p53 activation, thereby suppressing PEDV-induced apoptosis.

In conclusion, our results demonstrated that EP could inhibit multiple steps of the PEDV life cycle, including internalization, replication and release, and directly inactivate PDCoV infectivity. EP could inhibit PEDV-induced apoptosis through the ROS-dependent p53 signaling pathway (Figure 8). These results improve the understanding of the pathogenesis of PEDV infection and provide clues to the development of effective drugs against PEDV.

## Data Availability

Not applicable.
